# Implementing digital neuroscience in special-needs-teacher education: exploring student-teachers’ multifaceted learning outcomes related to teaching children with neurodevelopmental disorders

**DOI:** 10.3389/fpsyg.2023.1232315

**Published:** 2023-08-03

**Authors:** Rivi Frei-Landau, Etty Grobgeld, Raisa Guberman

**Affiliations:** ^1^The Rehabilitation Psychology Program, Faculty of Psychology, Achva Academic College, Arugot, Israel; ^2^The Neuro-padagogy Centre, Achva Academic College, Arugot, Israel; ^3^Department of Mathematics, Achva Academic College, Arugot, Israel

**Keywords:** digital learning, educational neuroscience, brain-based learning, neurodevelopmental disorders, inclusion

## Abstract

**Introduction:**

In recent decades, there has been increased use of neuroscience in teacher education, which refers to applying knowledge from brain science to teaching. Similarly, digital learning has been extensively integrated into teacher education, particularly in light of the COVID-19 pandemic. However, the benefits of assimilating educational neuroscience into special-education training—particularly using digital platforms–have yet to be examined. The current study explored the use of digitally-delivered educational neuroscience, related to neurodevelopmental disorders (ND), in teacher education, to gain insight into the learning outcomes alongside the contribution of the digital platform.

**Methods:**

Employing a qualitative approach, we recruited 193 student-teachers who learned a digital ND-related neuroscience course. Data collection included open-ended reflections, open-ended story questions and five focus groups – all of which were analyzed using content analysis.

**Results:**

Findings revealed a process involving four learning outcomes: understanding brain-based mechanisms of ND, enhanced empathy, extended perception of teachers’ professional role, and the design of pedagogical adaptations. The analysis also pointed out the various ways in which the digital platform facilitated these learning outcomes.

**Discussion:**

The study provides theoretical insight into the role of digitally-delivered educational neuroscience in the service of inclusion. It further discusses the practical implications of using digitally-delivered educational neuroscience in teacher education to promote an inclusive pedagogy and best practices.

## Introduction

In recent decades, there has been increased theoretical interest in the connections between the mind, brain, and education (Wilcox et al., 2021; Gola et al., 2022). As a result, a new field of research developed, which is referred to as educational neuroscience, neuropedagogy, or neuroeducation (Uden and Guan, 2022). This new field applies the findings of brain research to classroom teaching and learning, in general, and to teaching various K-12 teachers and students, in particular (Ansari et al., 2011; Brown and Daly, 2016; Thomas et al., 2019). Similarly, the education system had to adapt to the twenty-first-century developments in the digital field (Hsu, 2016; Geri et al., 2017; Mitsea et al., 2021, 2023). As such, the integration of digital technologies into schools and academia required the development of a new pedagogy in teacher education (Koehler and Mishra, 2008), which then could be adapted to suit the various content disciplines (Cui and Zhang, 2021). Recently, this need for a unique pedagogy became even more urgent, as the world coped with the transition to digital teaching due to the COVID-19 pandemic (Ching and Roberts, 2020; Frei-Landau et al., 2022a; Muchnick-Rozanov et al., 2022b; Hershkovitz et al., 2023). Although digital learning and neuroeducation have been explored separately, the use of digital educational neuroscience—particularly in the context of special education—has yet to be addressed. To address this research gap, the current study examined the outcomes of learning an Educational Neuroscience course focused on Neurodevelopmental disorders (ND) to student-teachers (STs) using a digital platform.

### Theoretical background

#### Educational neuroscience in teacher education

Educational Neuroscience is an emerging multidisciplinary field that integrates knowledge from behavioral sciences, cognitive psychology, neuroscience, and pedagogy, and it is defined as a subfield of education, neuroscience, and intelligence (Sousa, 2010; Knox, 2016; Vaughn et al., 2020). It is an interdisciplinary research field that seeks to translate research findings on neural mechanisms into educational practices (Uden and Guan, 2022). Although some argue that the core claim of educational neuroscience is that neuroscience can improve teaching in the classroom (Suresh et al., 2021), others claim that there are no current examples of neuroscience motivating new and effective teaching methods, arguing that neuroscience is unlikely to improve teaching in the future (Bowers, 2016). Hence, more research that unfolds this issue is needed.

Studies have noted that the teaching of Educational Neuroscience can lead to an in-depth understanding of processes of learning and memory (Goswrami, 2012); this understanding helps improve teaching, as well as classroom management strategies used by teachers (Brown and Daly, 2016; Howard-Jones et al., 2016), which in turn can lead to significant and effective in-class learning processes (Tokuhama-Espinosa, 2018). It was also argued that educational neuroscience may facilitate the design of interventions for improving executive functions (Cherrier et al., 2023). Considering these findings, teacher-education schools, colleges, and departments in academic institutions have begun to include courses in educational neuroscience in their curricula and explore their impact (Friedman et al., 2016, 2019; Guberman et al., 2022). For instance, Friedman et al. (2016) reported on pedagogical applications based on neuroscientific approaches, which can be applied to learning and teaching processes in the classroom. Examples of such pedagogical applications include teaching models; repetition, memorization, and memory solidification; previewing the lesson to enhance learning and teaching; using emotions to draw attention; harnessing the relationship between movement and learning; and increasing learning efficacy by organizing the learning processes to correspond to the learners’ circadian cycle. Another recent study (Guberman et al., 2022) showed that elementary-school mathematics teachers applied the neuroscientific theory of mathematical cognition in their classroom practices after participating in a neuroscience professional-development course. Specifically, they demonstrated that the teachers acquired knowledge about neuroscientific theories and integrated their newly acquired knowledge into their teaching.

However, the benefits of assimilating neuroscience into *special-education* curricula—particularly using digital platforms—have yet to be examined. Considering the ongoing trend of the inclusion policy (as reflected in amendment 11 of Israel’s Special-Education Act, 1988), a growing number of children with ND attend regular classrooms. As such, it is necessary to educate teachers on how to best interact with them and how to build best practices for them. It is further necessary to explore how exposure to educational neuroscience specifically related to children with ND affects teachers’ learning. Hence, the current study explored the benefits of integrating educational neuroscience related to NDs in special-needs-teachers’ training, to gain insight into the learning outcomes. However, this course was delivered digitally, using a novel teaching platform that has yet to be used in the context of teaching educational neuroscience.

#### Digital learning and teacher education

Digital learning enables teachers to teach students using a remote scenario (Carrillo and Flores, 2020). Digital platforms have become critical components in teacher education, as they allow for innovation and contribute to the learning process (Zadok and Meishar-Tal, 2015; Frei-Landau and Avidov-Ungar, 2022; Frei-Landau et al., 2022a). Specifically, the flexibility of digital tools helps teachers keep content up-to-date, elaborate on specific topics and address students’ individual learning needs (Heemskerk et al., 2011). In fact, long before the COVID-19 pandemic, digital learning was increasingly used in teacher education and scholars had explored its processes and outcomes (Kleinsasser and Hong, 2016). However, the COVID-19 pandemic highlighted the need to employ digital learning, in general, and in teacher education, in particular, in times of crisis (Ching and Roberts, 2020; Muchnik-Rozanov et al., 2022). A recent literature review of 134 studies, on the use of digital learning in teacher education during the pandemic revealed that one of the most researched topics was the effectiveness of the teaching-learning process and its outcomes (Carrillo and Flores, 2020).

Generally, studies that examined the efficacy and characteristics of teaching in a digital environment have indicated that many aspects of the learning experience improve (Luterbach and Brown, 2011). For example, it was found that an environment enhanced with innovative digital-based technologies promotes active learning, increases learners’ satisfaction with and pleasure from learning (Davidovitch and Yavich, 2017), and promotes significant and effective learning (Zadok and Meishar-Tal, 2015). Furthermore, research has also shown that digital learning has led to improved learning outcomes. However, it should be noted that at the same time, some studies emphasized the limitations of digital learning, claiming that the digitization of learning is more time demanding and hence is burdensome to the learner, which in turn might decrease the quality of the learning experience (Makransky et al., 2019).

Several advantages of the digital learning environment are mentioned in the literature. These include the option to work individually or cooperatively, exposure to materials and practice suited to the learner’s pace and times, immediate access to data and feedback, and the availability of visual media (Caspi and Blau, 2011; Forkosh-Baruch and Hershkovitz, 2012). Furthermore, the digital learning environment is supported by numerous instruments and study aids that can enhance the depth and breadth of learning, among them multimedia items, consultation forums, visual enrichment materials, videoconferencing, a shared whiteboard, document sharing, messages and blogs, as well as tools designed to assist learners with special needs (Irene and Athanasios, 2023), such as programs for voiced reading and input. These digital tools can be adapted for use with various media types (written, oral, or visual), which thus serve to enhance and vary the learning outcomes (Blau et al., 2018; Kurtoğlu and Karal, 2023). Learning outcomes are defined as the combination of knowledge and abilities acquired by learners after participating in a structured learning process, such as an academic course, and may include knowledge, skills, understanding, abilities, and attitudes acquired by the learners (Anderson et al., 2001; Wang et al., 2023).

#### Digital technology and special-needs education in the context of neurodevelopmental disorders

In recent years the use of technology and digitalization in special needs education has become more frequent, particularly in the case of neurodevelopmental disorders (ND). ND represent a new diagnostic category in the Diagnostic and Statistical Manual of Mental Disorders, 5th Edition (DSM-5; American Psychiatric Association, 2013), that includes a group of disorders that commonly begin in childhood and have a neurological basis and therefore require special needs education and adjusted teaching. ND include intellectual disability, attention-deficit/hyperactivity disorder (ADHD), autism spectrum disorder (ASD), specific learning disorders (i.e., learning disabilities, involving difficulties in reading, writing and arithmetic), motor disorders (such as Tics, Tourette etc.), among others (Francés et al., 2022). As such, scholars and policy makers representing an inclusive worldview have recently advocated for the implementation of inclusive pedagogy, which represents an approach that addresses interpersonal differences among learners with special needs without excluding them from the mainstream classroom (Spratt and Florian, 2015; Kurtoğlu and Karal, 2023). This innovative approach was developed in an attempt to address the needs of learners for differential support without treating them differently from their peers in the classroom (Florian and Beaton, 2018). Hence, it focuses on ways to promote—and train teachers to adopt—a teaching approach that includes all learners while addressing their unique needs.

Correspondingly, recent studies have explored the use of emerging technologies for facilitating best practices as well as teacher-student interactions, to provide educators with new opportunities for intervention, especially for children with NDs. For instance, Mitsea et al. (2022a,b,c) have used cutting-edge digital technologies to practice skills in special education, such as training individuals with autism and/or learning disabilities to use metacognitive skills (Mitsea et al., 2022a,d), arguing that soft skills and metacognition are inclusion amplifiers (Mitsea et al., 2021). Similarly, virtual reality games were used to practice meta-skills in special education (Drigas et al., 2022; Mitsea et al., 2023) and simulations were used to enhance empathy towards children with special needs (Frei-Landau et al., 2022b; Frei-Landau and Levin, 2022c). In this vein, the current study focused on exploring the learning outcomes of a digitally conveyed educational neuroscience course related to ND attended by STs. We further sought to understand whether and how the learning outcomes were related to the digital platform. As such, the following research questions were formulated:

### Research questions

1. How do STs perceive their learning outcomes in the process of learning and participating in the digital course in educational neuroscience focused on neurodevelopmental disorders?
2. In what ways did the digital platform facilitate STs’ acquisition of these learning outcomes?

## Methodology

### The study context

In 2016, a Center for Educational Neuroscience was established in a major teacher-education college in Israel headed by Professor Friedman. The Center launched the first pilot course for 26 students enrolled in a Master of Education program, and its outcomes were reported by Friedman et al. (2016). The current study was held at this Center for Educational Neuroscience.

#### The structure and contents of the digital neuroscience course

The first author, which is also the head of Special-Education department at the college, designed an online course (titled Brain, Learning, and Special Education) that dealt with neurological aspects of learning and teaching that can be relevant for use with special-education populations, mainly for children with learning disabilities, attention deficit disorder, autism spectrum disorder (ASD), or cerebral palsy (CP). Accordingly, emphasis was placed on aspects of neuroscientific knowledge applicable and relevant to learners with special needs, who are often included in mainstream classes in the school system. On a pedagogical level, the course dealt with neuroscientific aspects relevant to teaching and learning processes, while taking advantage of the variety of tools and teaching methods available in the digital learning environment, among them interactive assignments, cooperative learning using a digital forum, video clips, and exercises accessed through links to various Internet sites, use of multimedia items, and the combination of synchronous and asynchronous digital lessons.

**Figure 1:**
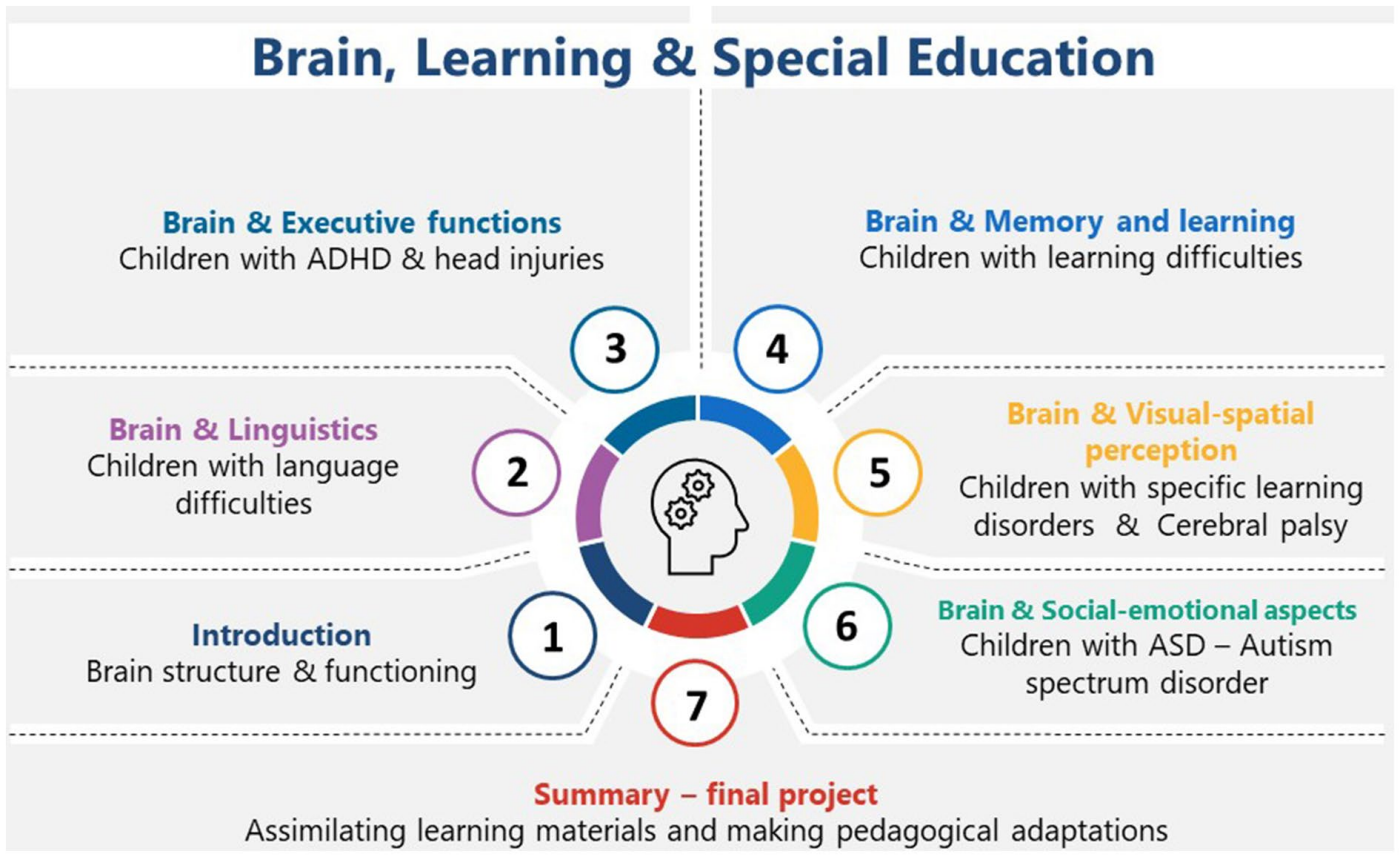
The structure of the course titled brain, learning, and special education, the study units, and the population of learners for which the unit and the corresponding brain functions are most relevant.

As regards the course contents, presented in Figure 1 below, the course included seven units, beginning with a preliminary introduction to the structure of the brain, its development, and functions. Each of the next six units dealt with a specific cognitive function, the brain regions that mediate this function, and the ND population that may be affected. Thus, for example, unit 2 dealt with unique diverse processes of the two hemispheres, their relation to language functions and to delayed language development or retrieval difficulties; unit 3 described the frontal lobes and executive functions and was discussed in the context of the needs of learners with attention deficit hyperactivity disorder (ADHD) or learners dealing with the effects of mild head injuries; unit 4 dealt with memory processes and was related to difficulties with work memory among children with learning difficulties and specific learning disorders; unit 5 dealt with visuospatial perception from a neural perspective and concerning spatial perception and analysis among learners with CP or learning disabilities; unit 6 dealt with the socioemotional functions and the regions that mediate them, and was related to the functioning of learners with ASD or with neurodevelopmental deficits resulting in social dysfunctions; unit 7 provided a review and summary of the course, including a final project. The current study describes the learning outcomes of STs who participated in this digital course, based on data collected over five academic years.

### The study design

Exploring a phenomenon, such as STs’ subjective learning experiences, calls for a qualitative methodology that captures the multifaceted nature of the phenomenon from the individual’s standpoint (Creswell and Poth, 2016). A case study design (Stake, 2005) was selected as a viable framework for exploring the research questions, as it allows for an in-depth investigation of the observed phenomenon within the particular professional learning environment at hand (in this case, the learning outcomes of learners exposed to digitally-delivered education neuroscience). Throughout the 5 years of data collection, the courses shared the same content, as shown in Figure 1. Thus, are all considered a case.

### Participants and data collection

The study participants included 193 students who attended the (specific) College of Teacher Education in Israel and—as part of their undergraduate study program—had been enrolled in one of the courses on Brain, Learning, and Special Education offered within the 5 years designated for data collection (2018–2022). The majority of the study participants were enrolled in the Department of Special Education (71%), while the remainder were enrolled either in the math-education department, in a national program for excellent students (11%); the early-childhood education department (9%); the English-teaching department (5%); or a different department (4%). Of the study participants, 87% were women and the remainder were men (a gender tendency common in education departments).

Data were collected using multiple data sources, to provide a comprehensive understanding of the explored phenomenon (Bogdan and Biklen, 1998), achieve trustworthiness through the triangulation of research methods, and enable cross-validity checks (Patton, 2002). Additionally, we conducted member checks—a frequently used method in qualitative inquiry (Frei-Landau et al., 2020c), to further support the study’s trustworthiness (Birt et al., 2016). The following modalities were used to collect the data:

1. *Participants’ post-course reflections*. At the end of the course, the STs handed in written reflections about their learning experience, the learning outcomes, and the manner in which the digital nature of the course affected the latter. They were asked to freely elaborate on their learning experiences and were encouraged to describe whatever issues they found relevant.
2. *An open-ended story question*: During this reflection, the STs were asked to answer two open-ended questions: The first requested that they elaborate on their learning insights and the second that they share a story related to their teaching experiences in schools (while learning the course) that reflects their learning from the educational neuroscience course. Overall, 193 reflections were collected.
3. *Focus groups*. As the last step of the research data collection, we held five focus groups (one each year) with participants who agreed to take part in it. During the focus group, the participants were requested to discuss their learning experiences and to respond to others’ comments about them. The focus group session lasted 45 min and was recorded and then transcribed.

### Ethics

The study was approved by the institutional ethics committee. The participants gave their informed consent. Personal information was concealed, thus ensuring participants’ anonymity; hence, when reporting the findings, pseudonyms are used. Participation was voluntary and participants were told they were allowed to refuse participation without risking any consequences.

### Data analysis

Braun and Clarke’s (2006) six-step inductive thematic analysis was used to analyze the interview contents. First, there was an initial reading and rereading of the transcripts, to become immersed in the data and to familiarize ourselves with the STs’ experiences. During this stage, each reader worked on her own to make a note of key statements pertaining to STs’ learning outcomes. In stage two, initial codes were generated separately by each coder across all data sets. This was followed by a discussion that sought to identify the most significant codes pertaining to teachers’ learning outcomes. This microanalysis was used to ensure that no important ideas or constructs were overlooked. In stage three, once all the data had been coded, the codes were classified into potential themes, through a process of comparison and contrast conducted to identify patterns and overarching themes. In the fourth stage, these themes were reviewed and refined to ensure internal homogeneity and external heterogeneity. The data were then reviewed once again, to ensure that the identified themes were comprehensive and properly supported and grounded in participants’ responses. In the fifth stage, the themes were yet again “refined and defined” (Braun and Clarke, 2006) through elicitation of the “essence” of each theme, and by giving them concise and mutually exclusive names. Finally, the sixth stage enabled the identification of those aspects that highlighted the studied phenomenon—STs’ multidimensional learning outcomes, which rendered four major learning outcomes derived from the learning process. It should be emphasized that data were coded and analyzed by independent coders (the authors), followed by recurrent brainstorming sessions. Cases of disagreement were discussed and settled through conceptual clarification and consensus.

#### Trustworthiness

While qualitative inquiry does not traditionally claim to produce “absolute truths,” it can instead strive to achieve what is known as “trustworthiness” (Korstjens and Moser, 2018) and “transferability” of the findings, which refers to the possible applicability of their results in other social contexts (Rodon and Sesé, 2008; Pratt and Yezierski, 2018). To this end, “investigator triangulation” was performed by two researchers during the coding, analysis, and interpretation of the data. Moreover, member checking was conducted, and participants’ comments were embedded into the findings. Finally, the authors engaged in critical self-reflection and examined their own preconceptions, feelings, and values (i.e., representing the principle of reflexivity) and the ways in which these might have affected their interpretations of the materials.

## Findings

### The learning outcomes (RQ 1)

**Figure 2:**
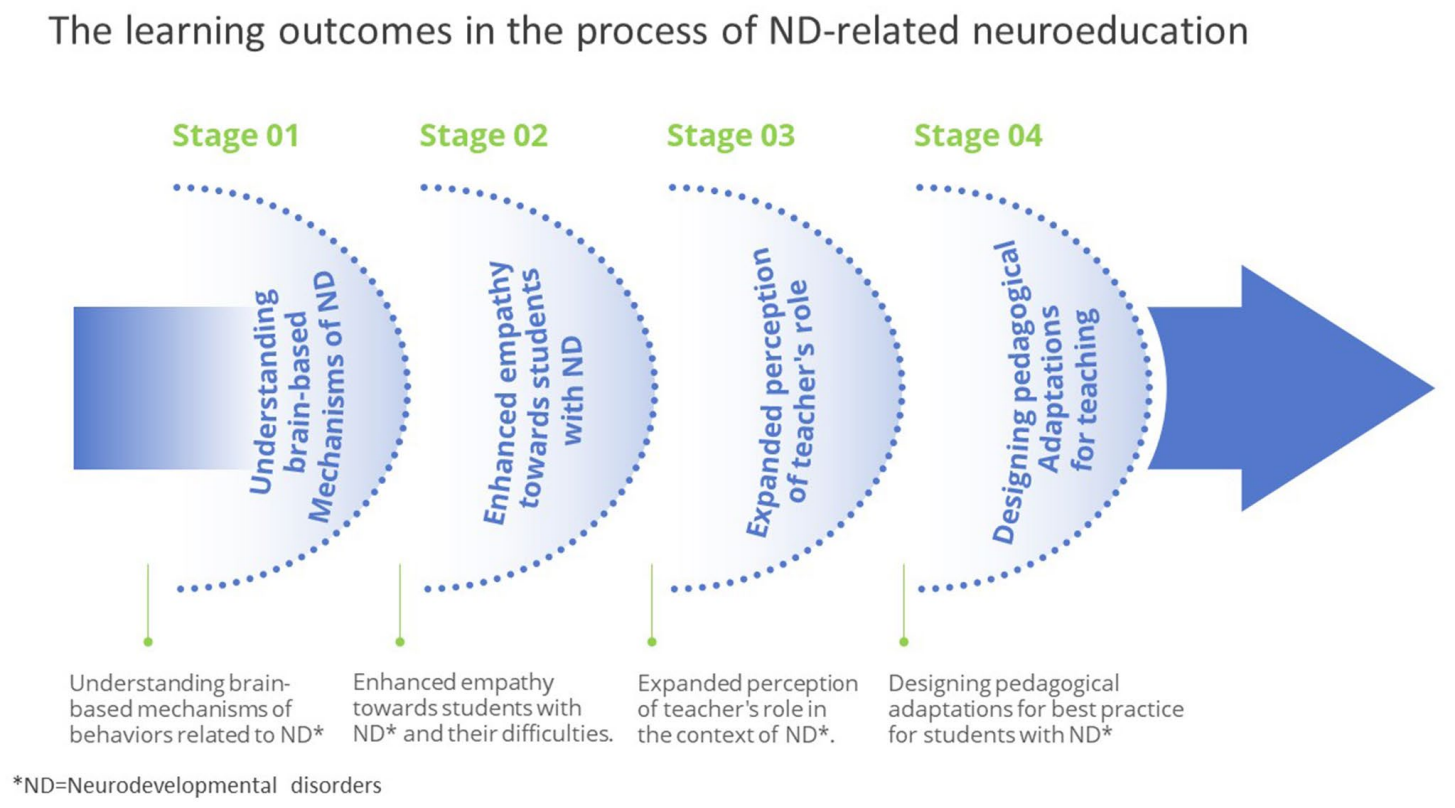
The study’s model—four learning outcomes.

Analysis of the findings indicated four types of learning outcomes reported by the STs following their participation in the digital course in educational neuroscience. All four learning outcomes involved both perceptual changes and emotional experiences. These outcomes were related to their role as teachers, and to their observations of their students’ experiences, as learners with ND. As displayed in Figure 2, these four learning outcomes evolved along a developmental process from “self to the other,” as follows: it begins with understanding brain-based mechanisms related to ND; followed by enhanced empathy towards students with ND and their resulting challenges. This cognitive and empathic shift was accompanied by a change in STs’ perceptions of their role as teachers. The learning process ended with them making practical pedagogical adaptations to their teaching techniques, according to the educational neuroscience knowledge they acquired.

The following section includes a description of the learning outcomes using the quotes from STs. All names are pseudonyms.

#### Understanding brain-based mechanisms related to ND

The participants described an enhanced understanding of brain-based mechanisms of ND, following their participation in the course, which eventually was experienced as the ability to better facilitate inclusion.

The course was enriching, and with five years of homeroom teaching experience, while learning some of the units, I suddenly saw before me some of my former students and especially one who this year is clearly having a hard time. Now I know how to name her difficulty. That gives me a good feeling … I simply have a better understanding now.

This is one example among many, in which the STs reported an enhanced ability to understand the difficulties and challenges of learners with ND, which gave them a sense of relief and was received as a fascinating experience:

Now I can explain much of the phenomena I see in the children with whom I work in special education … I have some experience working with children on the autistic spectrum…. The theoretical knowledge provides an explanation for what I am constantly witnessing and that is fascinating. For example, I can see that my student Tom (pseudonym) has difficulty predicting and understanding the behaviors of others. Now I understand the root of the social difficulties these students.

One of the STs who worked with children with behavioral problems explained that by understanding the brain mechanism, she is able to promote the child’s inclusion, which in turn leads to the child’s improved abilities. Thus, the process is respectful of the child and his or her needs.

As a homeroom teacher in a school for children with behavioral problems, this unit helped me understand that the root of their problem is deficient emotional regulation. For example, a child was frustrated and has a hard time regulating his reactions and consequently may demonstrate impulsive and violent behaviors. However, this is due to a delay or deficiency in the response of the prefrontal cortex to the stimulus that comes from the amygdala. Knowledge is power! In the context of teaching children with special needs, this knowledge can make the difference between momentary assistance and real progress and inclusion in society. Without understanding the theoretical background, one is more likely to make repeated mistakes and miss opportunities to help students advance.

#### Developing enhanced empathy towards children with ND and their difficulties

Developing empathy and acceptance were described by the participants as a major and significant learning outcome of participating in the course. Participants reported feeling greater empathy towards specific populations, such as children with late verbal development, ADHD, or learning disorders and claimed that this empathy emerges as the teacher becomes aware of the source of the child’s behavior in the classroom.

The findings presented in the current course were eye-opening and made us more aware, able to understand more, and be more accepting. In general, I think that I will be more understanding, for example, when a student with a learning disability is unable to organize or prioritize tasks.

Furthermore, it appears that there is a shift in the STs’ attributional thought patterns related to a child’s behavior, from an internal negative attribution (the child’s disruptive behavior is intentional) to an external attribution (the behavior is caused by a neurological deficit or difficulty).

Once we learned about the different parts of the brain and the differences (between the typical and atypical brain), our perception of students who are disruptive or behave unexpectedly can shift. We understand that sometimes the student has no control over these things…. For example, I understood the difficulties encountered by students with ADHD in terms of their executive functions, which can result in behaviors that antagonize teachers. But now I truly understand that it is a real challenge for them. I understand why the student may be disruptive or have difficulty and that it is not voluntary.

This perceptional change in thought patterns enabled the STs to accept the divergent behavior of learners with special needs and feel empathy toward them. As a result, they demonstrated greater patience, and their increased empathy motivated their desire to make the necessary pedagogical adaptations to help these children learn.

I wasn’t aware of the connection between ADHD and executive functions. The activity on the site with the clip showing children’s behaviors left a strong impression and made me understand a little more of what these students feel; it gave me a sense of what I can do to draw their attention. For example, giving brief and clear instructions gradually one stage after another; allowing them to move about as needed (because it is truly a necessity); believing in their ability and conveying this belief to them; and in general, demonstrating flexibility.

Another student specifically mentioned that this “eye-opening” that causes empathy results in better inclusion: “The information presented in the course was eye-opening and led us to pay closer attention, which enabled us to understand and better accept the students’ behaviors.”

#### Professional identity change—an expanded perception of the teacher’s role

The course participants described changes in their professional identity, with an emphasis on the expanded perception of the teacher’s role. They described changes related to two aspects: the first was a change in their role perception (which may have emerged following their new sense of empathy); specifically, they realized that the teacher’s role is not limited to conveying information but also includes establishing a learning atmosphere that is respectful and enabling.

I come from teaching mathematics as a discipline, through the program for excellent students, so I have no background in special education…. But I learned that as a teacher, I must make the material accessible to learners with attention deficit or learning disabilities and that this should be done calmly and patiently, avoiding cynicism at all costs, by nurturing a positive learning atmosphere in the classroom.

As seen, participants came to realize that the teacher’s role extends beyond teaching the material and also includes creating an enabling atmosphere in the classroom. The second aspect in which STs’ role perception shifted was in understanding that the teacher is obligated to make adjustments to the learners’ needs, using pedagogical tools and, furthermore, that this obligation is rooted in understanding the differences among learners and their needs. The participants’ reports indicated that their expanded role perception coincided with a greater motivation and willingness to make pedagogical adaptations in their teaching methods, to cater to the learner’s needs.

I feel that the material we learned helped me gain profound insight into the source of students’ learning disabilities and to internalize the understanding that as teachers, we are expected to adjust the assignments we prepare so that they are clear also to learners who have difficulty reading. Now, I not only know that this is necessary but I also understand why it needs to be done.

Another shift in their role perception came as the revelation that the teacher who understands the cause and character of the deficiency can have a positive effect on the learner’s self-image:

I understood that children who have difficulty with executive functions experience difficulties in getting organized, planning and solving problems, and in monitoring processes. All of these have a negative effect on their self-image. Hence, as teachers, we can have a strong influence on them, by adjusting how we respond to and address their difficulties. A helpful response can help increase the students’ confidence and self-image.

Another ST noted the following: “By trying to identify the student’s strengths and thought patterns, we can help leverage the strengths and create an experience of success; understanding how the brain works can help empower the learner.” This ST clearly states that understanding the workings of the brain helped her make the necessary adjustments in her attitude and responses, which can lead the student to experience success and empowerment.

#### Designing pedagogical adaptations for teaching

A large proportion of the participants’ reports referred to the need to produce practical pedagogical applications as one of the important outcomes of participating in this course.

I have learned to notice learners with organizational difficulties. I will be sure to teach learning strategies, and thus help students organize the material in their minds; I can help them set goals that they can accomplish and help them organize their activities within time constraints.

More specifically, the STs learned to identify symptoms of learning disabilities, which led them to attend to the adaptations that need to be applied: “For example, for children with difficulties in visual perception, I have come to understand how this learning disability affects the student. I now know how to adjust the learning process for these students.” A similar outcome reported was the ability to make adaptations to correspond to the needs of the various populations reviewed in the course (learners with ASD, learning disabilities, ADHD, etc.). Moreover, as a result of participating in this course, the participants described their decision to focus on students’ needs in the framework of their practicum, as the following example demonstrates: “Part of my job is to teach children with ASD. The unit about developing empathic abilities led me to work with my students on the issue of empathy.”

STs described various pedagogical adaptations that coincide with what they learned, as demonstrated in the following:

As part of our English lessons, we read the play All My Sons. My student had a very difficult time understanding the complex relationships described in that work; he managed to understand the plot but not what was motivating the characters or their behaviors. So I presented a version of the play in the form of a comic strip, which added a visual layer. In addition, we interpreted the work in a very concrete manner, without relating to the subtext.

Another ST described the pedagogical adaptations she made in her teaching as a result of the course:

I found that the section on the frontal lobes and the executive, monitoring and control functions that affect attention helped me a great deal in my work with children with attention deficit and behavioral problems. I emphasized the issue of self-organization and management, both in the classroom and during recess. I began by addressing pupils’ preparations before class, i.e., taking out their notebooks, pencils and pens, the sequence of required tasks, and then went through a variety of social situations in the classroom that occur daily and addressed proper ways to cope with each situation, e.g., waiting for your turn, accepting the rules of the game, solving problems without violence, etc.

### The role of the digital environment in promoting the learning outcomes (RQ 2)

The second research question examined the contribution of the digital platform to producing the learning outcomes. Analysis of the findings demonstrated that the STs reported a variety of ways in which the digital nature of the course enhanced the learning outcomes in each of the categories mentioned. Thus, for example, they reported using the study aids available on a website, the many study tools they could access, the availability of additional texts and visual clips, and the use of a forum for sharing with each other, as aspects that contributed to the development of the learning outcomes.

As follows are selected quotes demonstrating aspects of digital learning that promoted the learning outcomes:

#### Understanding brain-based mechanisms of ND

The tools on the website, all of the exercises and examples, as well as references to external websites, helped me gain an in-depth understanding. It suddenly made things clearer. I plan on keeping some of the course materials. The activities available online were very helpful.

As this example demonstrates, the STs described in detail the way the digital aids enhance their understanding of the material studied, i.e., the NDs. It appears that the online environment, the visual aids and the various exercises, helped the STs internalize their new knowledge.

#### Enhanced empathy towards students with ND

There were exercises on the website that increased my understanding…. For example, the figures … The examples provided…. The ability to share experiences from the practicum on the forum…. The demonstrations heightened my understanding of how my students feel, for example, the demonstration of how a child with a learning disability experiences the lesson was really eye-opening and meaningful. I understood that I need to exercise patience and sensitivity to accept and enable these students, to make them feel good and worthy of our belief in their abilities.

In this representative example, the ST notes that the digital demonstrations on the website provided an emotional experience that connected them with the experience of learners with NDs. This experience, in turn, aroused a new sense of empathy and understanding about the importance of accepting these students.

#### Expanding one’s role perception as a teacher

The activity on the website**—**regarding learners with difficulties in visual perception, provided a very impactful demonstration of the difficulties encountered by the learner and drove home my role as an educator in supporting their needs and finding effective teaching methods that work for them.

The video clip and the exercise that followed, about learners with attention deficit, really demonstrated the degree of pressure that these learners feel, and I have understood that the demands made by the teacher and the school system only increase this problem. I learned that as a teacher, I must allow them the time they need and not urge them to hurry.

As these examples clarify, the digital demonstrations not only helped develop STs’ sense of empathy towards their students with NDs but also raise their consciousness regarding their role as teachers, leading them to realize that it also includes helping students with special needs.

#### Designing pedagogical adaptations

I learned a lot about practice in the field and the tools that I can use as an educator…. For example, when we learned about learners with attention deficits, the questionnaires and exercises that we learned were very helpful for identifying the learners’ precise problems. I also liked what we learned about addressing these difficulties: the use of organizational charts for morning and evening activities. I will definitely use these and print them out for my work with the students. It taught me the type of things I need to notice in order to mediate verbally the goal or objective (as shown in the demonstration), and perhaps even to seek other ways to help them**—**to be creative about it.

## Discussion

The current study examined the learning outcomes of a digitally-delivered educational neuroscience course related to children with ND. The study’s findings highlight four types of learning outcomes reported by the STs following their participation in the digital course in educational neuroscience. These four learning outcomes evolved along a developmental process from oneself to the other, as follows: it begins with understanding brain-based mechanisms related to ND, followed by enhanced empathy towards students with ND and their resulting difficulties, which is then accompanied by a change in STs’ perceptions of their role as teachers, and ends with them making practical pedagogical adaptations to their teaching techniques, according to the educational neuroscience knowledge they acquired. Hence, the current study affords an integrative view of the learning outcomes. The study also highlights the benefit of the digital platform in this context, by showing the variety of ways in which the digital nature of the course enhanced the learning outcomes in each of the categories mentioned. In this vein, this study further strengthens prior claims regarding the suitability of cutting-edge technologies for promoting best practices in special education. For instance, Mitsea et al. (2022a,b,c) have used cutting-edge technologies to practice skills in special education training, such as promoting metacognitive skills among individuals with autism (Mitsea et al., 2022a) or learning disabilities (Mitsea et al., 2022d). Similarly, Mitsea et al. (2022a,b,c,d) used virtual reality games to practice metacognitive skills among children with special education needs.

### Digital neuroscience in the service of STs’ professional development

The findings of the current study correspond with previous findings in this field, showing that educational neuroscience may affect teachers’ professional development perceptions (Hachem et al., 2022) and contribute to best practices (Luque-Rojas et al., 2022). Thus, for example, in a previous study that interviewed teachers about their views regarding the relevance and advantages of educational neuroscience (Hook and Fara, 2012), the participants reported their interest and enjoyment of being intellectually involved in an innovative field and mentioned three learning outcomes: (a) enhanced self-confidence, professional control and authority; (b) a changed perception of and increased patience and empathy towards challenging students, in light of their understanding of the neurological processes that affect these students; and finally, (c) professional satisfaction and an improved self-image, caused by their understanding of the important role played by the teacher, namely, nurturing the mind and consciousness of the learner. Some of these learning outcomes were also identified in a sample of 80 graduate students who participated in a face-to-face course on educational neuroscience (Friedman et al., 2019). Specifically, the following four major themes emerged from the study’s analysis of the participants’ reports: (1) It is essential to apply basic neuroscientific knowledge in contemporary teaching practices. (2) Neuroscientific knowledge provides a conceptual underpinning to teachers’ commonly used pedagogical practices, which enhances teachers’ professional competence and confidence. (3) Knowledge of neuro-processes enables teachers to devise different pedagogical approaches and methods. (4) Gaining an understanding of different learners’ neuro-functions can help teachers choose alternative approaches that are better suited to their students’ needs (Friedman et al., 2019). Hence, the current study reinforces the findings of the two aforementioned studies while also honing our understanding regarding the broader role of teachers of students with NDs, which requires both a shift in teachers’ perceived professional identity and the inclusion of empathy. However, in contrast to the previous findings, which emerged from studies involving courses taught face-to-face, the current study involved teaching via digital platforms, thus amplifying the implications of the previous studies, while demonstrating the benefits of using cutting-edge technologies.

### Digital neuroscience in the service of inclusion

Inclusive pedagogy, which stems from an inclusive worldview, represents an approach that addresses interpersonal differences among learners in a way that refrains from either labeling learners with special needs or excluding them from the mainstream classroom (Spratt and Florian, 2015). This innovative approach was developed in an attempt to address the needs of learners for differential support without treating them differently from their peers in the classroom (Florian and Beaton, 2018). As a result, this approach focuses on ways to promote teaching that includes all learners while addressing the unique needs of each. Furthermore, the current study’s findings indicate that STs’ exposure to knowledge about the brain’s functioning and mechanisms in learners with special needs motivated them to adopt and promote an inclusive pedagogy.

An interesting finding is related to the process of STs’ attributions. Studies indicate that understanding teachers’ cognitive perceptions regarding inclusion is essential. Specifically, teachers’ beliefs, knowledge and attitudes about the mental functions that lead to special needs were found to play a major role in their classroom practices (Sherman et al., 2008). One such cognitive domain is teachers’ attributions (interpretations) regarding the misbehaviors of students with ND.

The attributional theory (Weiner, 2000) conceptualizes one’s interpretation of oneself and others’ behaviors. It focuses on the causal explanations that individuals give when judging an event. These attributions are made along three dimensions: the locus of control, which addresses the question of who/what is responsible for the event (internal, external); stability, which assesses whether the situation will persist (stable, unstable); and controllability, which asks: Is it possible to control the event? (controllable, uncontrollable). Weiner (2000) has claimed that when an undesirable event is perceived as internal and controllable, individuals tend to place blame upon the individual, and thus perceive him/her as deserving an angry response, punishment and little sympathy. In contrast, perceiving the cause as external and uncontrollable (i.e., an illness, or a disorder) is associated with viewing the person as deserving of sympathy.

Applying the attributional theory to one’s attributions for students with ND-related misbehaviors is interesting. For instance, studies have found that parents of children diagnosed with ADHD who attributed their misbehaviors to an internal locus, with stable and controllable causes (i.e., “He misbehaved on purpose. He always does that”), tended to display harsher and more negative discipline methods—which, in turn, predicted escalation in child’s problem behaviors (Johnston and Ohan, 2005). Eventually, this may result in endorsing a more punitive, harsh and criticizing educational strategy (McAuliffe et al., 2009), impeding teachers from helping students to manage their behaviors (believing that it is under their control) and affecting teachers’ implementation of helpful classroom practices (Mikami et al., 2019). This state of affairs often results in an escalation of undesirable behaviors; hence, these attributions are an important target for exploration, awareness and intervention. Correspondingly, discerning teachers’ attributions when facing ADHD-related (or any other ND-related) behaviors in the classroom is essential (Mikami et al., 2019). The current study indicated that exposing STs to ND-related neuroeducation has the potential to shift their attributions, thus promoting better treatment and inclusion. This echoes the findings of a study that showed that using educational neuroscience in teacher training may facilitate teachers’ understanding of neuromyths (Arslan et al., 2022).

In the same vein, a major finding that is particularly important in the context of inclusion is related to STs’ report of increased empathy regarding the difficulties experienced by learners with ND. As mentioned, participants reported feeling more empathic toward students with ND-related difficulties. This enhanced empathy was related to their understanding of the neurological mechanisms, which was facilitated by the use of digital media and technology. This corresponds to a previous finding, which demonstrated that informed use of relevant media has the potential to enhance one’s empathy towards others (Batson and Ahmad, 2009).

Ultimately, it appears that STs have experienced both cognitive, perceptual and emotional changes. Avni and Rotem (2013) claimed that learning becomes significant “When it is important, valuable, and meaningful to the learners and corresponds to their conceptual, cognitive, and emotional world, such that the learning experience fashions the learners’ reality, personality, skills, development, and future.” Accordingly, it may be assumed that it is likely that the changes reported by the learners in this study, which were related to both their emotional and cognitive worlds, would be internalized by the learners and applied in their future work as teachers.

### Limitations and implications

Longitudinal studies over several years could further our understanding of the practical implementation of the knowledge gained over time, demonstrating whether these learning outcomes develop and/or change over time and with increased experience. In addition, it should be noted that all self-report measures may have been influenced by social desirability bias. Nevertheless, we believe that the triangulation of data sources helped minimize the chance of bias as much as possible.

Future research may opt to explore whether these learning outcomes manifest similarly or differently at teachers’ different career stages and whether personal experience plays a role in this process. Finally, future research may be conducted among learners of various cultures and minority groups, as participants’ background was found to be an essential factor affecting their adjustment in managing complex situations (Frei-Landau et al., 2020a,b).

In conclusion, the current study’s findings demonstrate that using educational neuroscience in teacher education promotes and facilitates inclusion and that in this context, the digital platform has a beneficial role. Thus, this study contributes to the ongoing conversation about ways to advance STs’ acceptance and use of inclusive pedagogies and best practices. This is imperative, given the current worldwide concern with the pursuit of social justice, manifested in the trend of including children with NDs in mainstream classrooms. The study also contributes to the ongoing debate on the benefit of implementing educational neuroscience in teacher education.

## Data availability statement

The raw data supporting the conclusions of this article will be made available by the authors, without undue reservation.

## Ethics statement

The studies involving human participants were reviewed and approved by Achva Academic College Ethics Committee (2018_76), Israel. The patients/participants provided their written informed consent to participate in this study.

## Author contributions

All authors listed have made a substantial, direct, and intellectual contribution to the work and approved it for publication.

## Conflict of interest

The authors declare that the research was conducted in the absence of any commercial or financial relationships that could be construed as a potential conflict of interest.

## Publisher’s note

All claims expressed in this article are solely those of the authors and do not necessarily represent those of their affiliated organizations, or those of the publisher, the editors and the reviewers. Any product that may be evaluated in this article, or claim that may be made by its manufacturer, is not guaranteed or endorsed by the publisher.
